# Recognition of Immune Response for the Early Diagnosis and Treatment of Osteoarthritis

**DOI:** 10.1155/2015/192415

**Published:** 2015-05-03

**Authors:** Adrese M. Kandahari, Xinlin Yang, Abhijit S. Dighe, Dongfeng Pan, Quanjun Cui

**Affiliations:** ^1^Department of Orthopaedic Surgery, University of Virginia School of Medicine, Charlottesville, VA 22908, USA; ^2^Department of Radiology and Medical Imaging, University of Virginia, Charlottesville, VA 22908, USA

## Abstract

Osteoarthritis is a common and debilitating joint disease that affects up to 30 million Americans, leading to significant disability, reduction in quality of life, and costing the United States tens of billions of dollars annually. Classically, osteoarthritis has been characterized as a degenerative, wear-and-tear disease, but recent research has identified it as an immunopathological disease on a spectrum between healthy condition and rheumatoid arthritis. A systematic literature review demonstrates that the disease pathogenesis is driven by an early innate immune response which progressively catalyzes degenerative changes that ultimately lead to an altered joint microenvironment. It is feasible to detect this infiltration of cells in the early, and presumably asymptomatic, phase of the disease through noninvasive imaging techniques. This screening can serve to aid clinicians in potentially identifying high-risk patients, hopefully leading to early effective management, vast improvements in quality of life, and significant reductions in disability, morbidity, and cost related to osteoarthritis. Although the diagnosis and treatment of osteoarthritis routinely utilize both invasive and non-invasive strategies, imaging techniques specific to inflammatory cells are not commonly employed for these purposes. This review discusses this paradigm and aims to shift the focus of future osteoarthritis-related research towards early diagnosis of the disease process.

## 1. Introduction

Osteoarthritis (OA) is a painful and debilitative joint disease that commonly affects the hand, hip, and knee joints of aging adults. Disease progression is a leading cause of hospitalization and ultimately requires joint replacement surgery which costs the US healthcare industry over $42 billion in 2009 for the hip and knee joints alone [1]. Clinical OA affects up to 30 million Americans including one-third of seniors aged 65 or older and 13.9% of all adults at least 25 years of age [2]. While disease-modifying antirheumatic drugs (DMARDs) have been identified for rheumatoid arthritis (RA), an inflammatory joint disease often studied and characterized in comparison with OA, similar therapy for OA has yet to be identified [3, 4]. The classical definition of OA as a wear-and-tear, noninflammatory disease has recently transitioned to an inflammatory disease lying on a spectrum between normal control and RA. Despite the fact that the immune system plays a significant role in both diseases, DMARDs effective in the treatment of RA, including tumor necrosis factor *α* (TNF*α*) and interleukin-1 (IL-1) inhibitors, have so far proven unsuccessful in slowing disease progression and clinical deterioration of OA patients. This paper will characterize the key players in OA pathogenesis and identify disease-modifying therapeutic strategies which could be reasonably accommodated in the setting of a prevalent, high-morbidity, and costly disease in the United States of America.

Recent research has established that multiple cells, cytokines, chemokines, complement, and other aspects of the immune system are involved in the pathogenesis of OA, with the roles of integral cells and proteins summarized in Tables 1 and 2, respectively. There exists a continuum of inflammation along the spectrum of normal, OA, and RA, with progressive increases in cytokines and other mediators of inflammation along with leukocyte infiltration [5]. OA pathogenesis is multifactorial and complex with evidence pointing towards unique phenotypes and seemingly discrete stages: early, intermediate, and late. Numerous pathways exist and not all may be implicated in specific joints or individuals, but all eventually lead to the endpoint of joint degeneration.

## 2. Immune Response

### 2.1. Early Innate Response

Both the innate and adaptive immune systems have been implicated in OA pathogenesis, but of particular interest is the role of the innate immune system in early OA. Pathogenesis begins with trauma to the joint, which may constitute repetitive microtrauma accumulated throughout a lifetime or a major traumatic event such as articular fracture. Trauma to the joint is absorbed by subchondral bone [6] and joint-associated fat pads [7], respectively. Subchondral bone releases cytokines while the fat pads release adipokines such as leptin, resistin, adiponectin, visfatin, and chemerin [7]. Although the role of adipokines in OA remains to be conclusively elucidated, many studies have implied that they may act as chemokines and increase matrix-degrading enzymes matrix metalloproteinase (MMP) and a disintegrin and metalloproteinase with thrombospondin motifs (ADAMTS) [7–9], nitric oxide synthase (NOS) [10], Toll-like receptor (TLR) [7], and other cytokine production [7, 11]. Additionally, joint-associated fat pads are innervated by C-fiber neurons which release substance P, thereby increasing pain sensitivity, proinflammatory cytokine production, and vascular permeability [12, 13]. This series of events leads to the release of damage-associated molecular patterns (DAMPs), or alarmins, from the extracellular matrix (ECM) by both direct trauma and the action of MMPs and ADAMTS, as well as from neutrophils and monocytes. DAMPs stimulate TLRs on macrophages and chondrocytes, inducing a strong upregulation of catabolic markers (MMPs 1, 3, 9, and 13, IL-6, IL-8, and monocyte chemotactic protein 1) and cytokines TNF*α* and IL-1*β* by way of NF*κ*B activation, which is the master regulator in immune response [14–16]. This chronic activation of TLRs leads to their upregulation in chondrocytes [15] and increased sensitivity [16].

**Figure 1:**
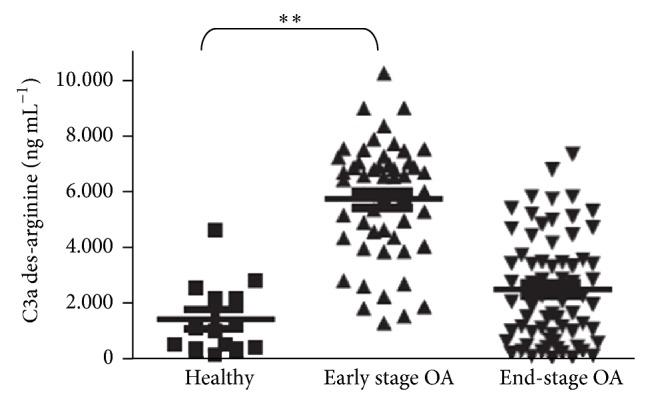
Complement synovial infiltration in the early pathogenesis of OA. ELISA quantification of C3a des-arginine in synovial fluid of healthy (*n* = 14), early-stage OA (*n* = 52), and end-stage OA (*n* = 69) patients. C3a des-arginine is a carboxypeptidase-cleaved, stable form of C3a that is generated from C3 during activation of the complement cascade. ^**^ *P* ≤ 0.01 by one-way analysis of variance (ANOVA) and Dunnett's post hoc test (reproduction of image with permission and modified caption from Wang et al. [17]).

The actions of complement are further demonstrating the significant role played by the innate immune system in early OA. Wang et al. reported that complement expression and activation were abnormally high in OA synovium, especially in early OA, seen in Figure 1. Additionally, the membrane attack complex (MAC, C5b-9) was present surrounding chondrocytes in late OA [17]. MAC directly damages the cell membrane but also stimulates MMP, ADAMTS, and chemokine production in chondrocytes, leading to increased chondrocyte destruction, catabolism of cartilage, and leukocyte infiltration. MMPs release components of the extracellular matrix, such as fibromodulin and aggrecan, which further induce MAC formation. To further assess the role of complement, Wang et al. knocked out C5 in mice and observed that, compared to C5^+^ controls, the C5^−^ mice showed no significant synovitis or cartilage loss 8–12 weeks status post (s/p) medial meniscectomy, a surgery that can induce OA. Furthermore, C6^−^ mice developed about half the synovial degeneration as C6^+^ mice s/p medial meniscectomy. CD59, a MAC inhibitor, was also knocked out in another mouse model, and these mice developed more severe OA compared to controls [17]. In another study, Busby Jr. et al. found that inhibiting C1s, a serine protease involved in the initiation of the classic activation pathway, promoted favorable joint architecture in dogs. One mechanism by which C1s exerts its effects is by cleaving chondroprotective IGFBP-5 [18].

Other innate immune cells have also been found to play a role in pathogenesis. NK cells have been found in the synovium of OA patients, in one study exhibiting a CD16^+^CD56^+^ phenotype both with and without granzymes A and B [19]. Granzyme A and B expression correlates with cytolytic potency* in vitro* [19]. In another study, NK cells were identified within OA synovia with a CD16^−^CD56^+^ phenotype without granzyme expression. Additionally, these cells demonstrated poor production of interferon *γ* (IFN*γ*), a cytokine central to osteoclastogenesis, upon stimulation* in vitro* [20]. In yet another study, granzymes A and B could be identified in the synovia from OA, RA, and reactive arthritis patients [21]. These findings imply that, in OA joints, NK cells can be of an active, cytolytic phenotype, or of an exhaustive, postactivation versus immunoregulatory phenotype. Granzymes A and B, exclusively produced by cytolytic lymphocytes, were identified both intracellularly in NK cells and in the synovia of OA patients [19, 21]. While granzyme presence in the synovium could be explained by T cells, the exclusiveness of this is unlikely. The production and release of granzymes [19, 21] support the notion of an activation/postactivation phenotype theory of NK cell involvement [20]. Of note, Huss et al., who identified mostly CD16^−^CD56^+^ NK cells negative for granzymes and suggested that NK cells are of the immunoregulatory phenotype [20], performed their analysis on patients undergoing primary or revision joint replacement, indicative of late OA patients. Concordantly, IFN*γ* production and degranulation of NK cells were significantly lower after* in vitro* stimulation of synovial tissue taken from revision versus primary joint replacement patients (degranulation of 2% and 7%, resp., *P* < 0.05) [20]. The decreased sensitivity of synovial NK cells to stimulation in revision versus primary joint replacement patients demonstrates evidence for an exhaustive NK cell phenotype in late OA. Most likely there is a combination of both activating and immunoregulatory roles played by NK cells in OA pathogenesis.

Mast cells have been identified in the synovium of OA patients [22–24], and in one study their counts were found to have a positive correlation with total cellular infiltrate (*r*_*s*_ = 0.82, *P* = 0.0141) [19]. Interestingly, no correlation between ESR and mast cell count or total cellular infiltrate was found, suggesting only local effects in the joint microenvironment inconsistent with markers of systemic inflammation or disease process [22]. This point is a major barrier to diagnosing and monitoring OA and is expounded upon in later sections. Mast cells are a potent regulator of vascular permeability, and they may play a crucial role in leukocyte recruitment to OA joints. Degranulated mast cells have been found in OA synovium [23], and Buckley et al. discovered a selective expansion and higher ratio of mast cell tryptase phenotype in OA synovium, a phenotype consistent with degranulation [24].

While the significance of neutrophils in synovial disease is well characterized in RA, the role of neutrophils in OA is relatively unknown. Neutrophils are found in varying levels in the synovium of OA patients but generally are found only in small numbers if present at all [5]. However, human neutrophil peptides 1–3 (HNP1–3), *α*-defensins, were found in the synovial tissue of both OA and RA patients in one study [25]. Interestingly, stimulation with TNF*α* led to the inhibition of HNP1–3 levels in the synovium of OA patients but not RA patients. The authors concluded that this was most likely due to desensitization of TNF receptors in RA synovia. Paired with the finding that HNP1–3 stimulates macrophages to release TNF*α* [26], the authors concluded that TNF forms a negative feedback loop with HNP1–3 [25]. If HNP1–3 release does precede the actions of TNF*α*, this would suggest that neutrophils play a role in early OA pathogenesis, as TNF*α* is a central mediator of the disease process. In another study, neutrophil gelatinase-associated lipocalin (NGAL) was found in complex with MMP-9 in OA synovia. NGAL served to decrease degradation of MMP-9 [27], found to be the predominant gelatinase in actively resorbing cartilage [28]. In the presence of NGAL-MMP-9, increased levels of glycosaminoglycan were released from cartilage explants* in vitro* [27]. The role of the innate immune response in early OA pathogenesis is summarized in a stepwise fashion below.

- Trauma to the joint is absorbed by subchondral bone and fat pads.
- Cytokines, MMPs, and ADAMTS are released.
- Direct trauma and MMP/ADAMTS activity release DAMPs which stimulate TLRs.
- TLR activation stimulates NF*κ*B, the release of cytokines (mainly TNF*α* and IL-1*β*), macrophages, complement, catabolic pathways in chondrocytes, other innate immune cells, and ultimately the adaptive immune response.
- Chronic cascading increases TLR expression and receptor sensitivity, further increasing inflammation.

### 2.2. Adaptive Response

Actions of the innate immune system inevitably lead to activation of the adaptive immune system, increasing inflammation and damage to the joints. TNF*α* and IL-1*β* are the dominant and most abundant cytokines implicated in OA [5]. They act independently of each other and additively to shift synovial tissue homeostasis towards catabolism [29, 30]. Mechanisms of this shift include increased resorption and inhibition of proteoglycans in cartilage, production of MMPs and chemokines, endothelium activation, and induction of apoptosis in chondrocytes [31, 32]. This leads to increased macrophage and CD4^+^ T cell infiltration, blood vessel formation by increased vascular endothelial growth factor (VEGF), and increased cyclooxygenase-2 level [33]. Macrophages and T cells, specifically of the CD4^+^ Th1 subtype [34, 35], are the most abundant cell types found in the synovium of OA patients [5, 36]. Their activation initiates a repetitive cascade of events, activating both the innate and adaptive immune systems, and this propagating inflammation destroys increasing amounts of cartilage, decreasing function and increasing morbidity. T cells are responsible for enhanced stimulation of macrophages and the activation of B cells. Autoreactive B cells further damage cellular integrity and increase inflammation by producing autoantibodies specific for cartilage cell surface proteins such as osteopontin and collagen. Elevated titers of these autoantibodies were found in the sera from OA patients compared to controls [31]. The adaptive immune response is summarized in stepwise fashion below.

- Cytokine release and increased vascular permeability lead to T-cell infiltration.
- T cells release chemokines and cytokines including IFN*γ*, further stimulating macrophages.
- Antigen presentation activates B cells.
- B cells release IL-6, increasing acute phase reactants, and produce autoantibodies causing direct damage to cartilage.
- Lymphocyte and macrophage activation in the joint microenvironment lead to a chronic, relapsing course of inflammation.

## 3. Early Diagnosis and Treatment

### 3.1. Imaging Techniques

Anatomic imaging techniques, such as radiography and magnetic resonance imaging (MRI), are currently used for epidemiological studies and clinical trials [37, 38]. Plain radiography is the traditional approach to monitoring progression of disease by clinicians; however, the drawbacks of this approach are apparent: insensitivity to change, nonspecificity, susceptibility to measurement error due to change in positioning, and inability to detect early stages of disease [39, 40]. MRI is regarded as sensitive, valid, and reproducible in that it can assess abnormalities of the whole-joint structure including cartilage degeneration [41], subchondral bone marrow lesions [42, 43], meniscal defects [44], and joint effusion and synovitis [45]. However, even MRI is not sensitive enough to detect the early immune cell infiltration of joints in OA, as inflammation far precedes cartilage destruction marked by radiographic change [46].

There is a substantial need to develop imaging techniques that can visualize the activity of the disease process itself, rather than measure structural changes that are a result of the disease process [47]. In this regard, a few reports have been published on the use of functional nuclear imaging techniques, such as positron emission tomography (PET) and planar or single-photon emission computed tomography (SPECT), for monitoring the inflammatory process of OA [48]. ^18^F-2-Fluoro-2-deoxy-D-glucose and ^111^In-diethylene triamine pentaacetic acid-folates have been explored as imaging tracers for OA because of respective increased metabolism of glucoses and elevated expression of folate receptors in activated immune cells [49]. Although these tracers have demonstrated some promise in clinical trials as well as in experimental OA models, they are likely not in use due to the lack of an inflammation-specific window of opportunity for imaging.

Alternatively, formyl peptide receptor (FPR) is primarily expressed on activated leukocytes as a defense mechanism to detect and trigger an immune cell response to inflammation caused by infections in a time and concentration dependent manner [50]. In the past years, based on a FPR-specific binding peptide, cFLFLF, we have successfully utilized the cFLFLF-PEG modules to build PET (1,4,7,10-tetraazacyclododecane-1,4,7,10-tetraacetic acid-^64^Cu, also known as DOTA-^64^Cu), SPECT (^99m^Tc), and optical (cyanine-5 and cyanine-7) imaging probes and exhibited excellent imaging in a variety of animal inflammation models [51–55]. The cFLFLFK-Cy-7 probe is now commercially available (Kerafast, Inc.) and cFLFLF-based probes have been developed enthusiastically for animal imaging by the broader research community [56–58].

**Figure 2:**
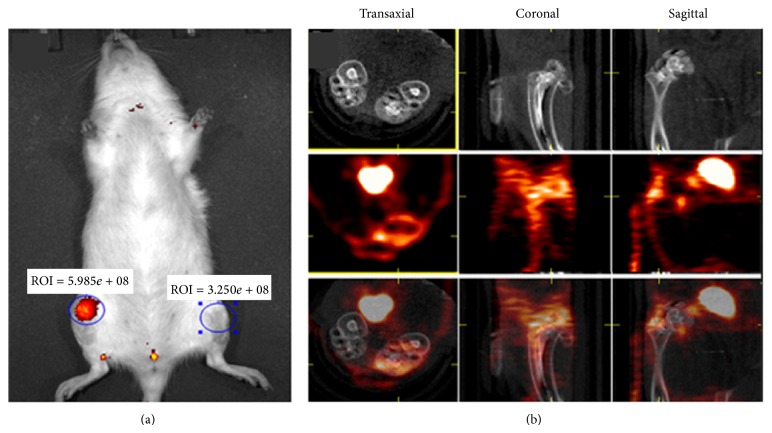
*In vivo* imaging of inflammation with two cFLFLF-derived probes in the rat knee joints treated with (right knee) or without (left knee) monoiodoacetate (MIA). (a) CFLFLF-PEG-Cy 7 probe with animal back down, at day 5 after MIA injection; (b) cFLFLF-PEG-DOTA-^64^Cu with animal back up, at day 5 after MIA injection (upper column: micro-CT; middle column: micro-PET; lower column: fused).

We are currently exploring if a cFLFLF-based SPECT imaging approach is feasible to monitor aseptic inflammation with a particular interest in OA. To this end, an acute model was created by intra-articular injection of monoiodoacetate for near-infrared fluorescence (NIRF) or PET imaging of inflammatory cells during OA development in rat knee joints. As shown in Figure 2, the inflamed joints were well imaged by either a NIRF probe cFLFLF-PEG-Cy 7 (Figure 2(a)) or a PET probe cFLFLF-PEG-DOTA-^64^Cu (Figure 2(b)). If available in the clinic, use of this SPECT technique can facilitate early detection and monitoring of the recruitment of innate leukocytes during OA development, allow correct characterization and diagnosis to direct early appropriate intervention, and improve long-term outcomes in OA patients [59].

**Figure 3:**
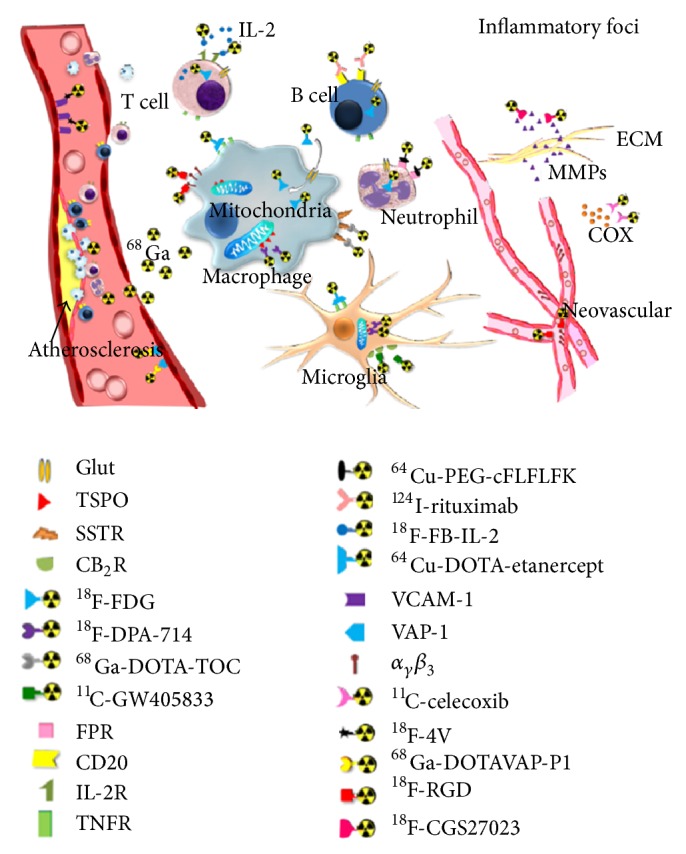
Inflammatory biomarkers in PET imaging (reproduced with permission from Wu et al. [99]).

As a caveat, FPR expression in fibroblasts and mesenchymal stem cells (MSCs) has been demonstrated [60, 61]. However, these MSCs and fibroblasts likely serve to repair tissue, initiate tissue remodeling, and mediate leukocyte infiltration in response to the acute chemotactic stimuli of formyl peptide, thereby still reflecting early changes on imaging. The actions of fibroblasts are noted in Tables 1 and 2, respectively. MSCs decrease inflammation, and overexpression of FPR in these cells is currently being studied as potential therapy in chronic disease such as cystic fibrosis [61]. Additionally, FPR ligands have been shown to decrease inflammation in joints and have even been suggested as potential therapies for RA [62]. Regarding the utilization of FPR as an imaging target in early OA, the actions of MSCs, fibroblasts, and FPR ligands, while noteworthy, should have little to no effect or have not yet been discovered. Probes for many cell types and mediators of inflammation mentioned in this paper are displayed in Table 3 and Figure 3.

### 3.2. Biomarkers

To date, many barriers exist in identifying biomarkers reflective of OA severity; histochemical findings have yet to be linked to clinical traits such as pain and function. Foremost, as evidenced in the next section, inflammation in OA is not only local but also systemic, making standard systemic measurements from individual to individual difficult. The confounding factors in systemic inflammation are immeasurable: age, genetics, diet, activity, kidney function, liver function, weight, and other comorbidities to name a few [63]. Numerous biomarkers have been thought to show promise in recent studies, such as serum cartilage oligomeric matrix protein and urine C-terminal cross-linked telopeptide type II collagen levels, but these are nonspecific to cartilage [63, 64]. Complicating the lack in specificity of inflammatory biomarkers is that measurements in OA patients are drawn once disease is already established. The ability is compromised to determine baseline patient values, cut-off values distinguishing normal from abnormal, and markers that are pathological rather than released naturally or concurrently. Another major barrier limiting the identification of both biomarkers and effective treatment is the unfortunate discrepancy between* in vitro* and* in vivo* studies. Decreasing specific mediators of inflammation has thus far not led to improved pain score or prognosis* in vivo*.

For these reasons testing biomarker levels in synovial fluid seems appropriate. However, the natural microenvironment between individual joints varies, making standard measurements difficult to implement [63]. This is evidenced by the unique infrapatellar fat pad of the knee, which greatly contributes to OA pathogenesis by way of adipokine release. Additionally, extracting synovial fluid is restricted to the larger joints and carries risks as compared to drawing blood. Finally, different phenotypes of disease presumably involve diverse biomarkers, pathways, and sequelae [63]. As evidenced by Table 1, T cells [34, 65] and NK cells [19, 20, 66] have been shown to possess exhaustive and chronic phenotypes, respectively, in late versus early disease, demonstrating that early disease is the primary mechanism responsible for changes in the joint microenvironment, underlining the importance of identifying these changes.

### 3.3. Hurdles to Treatment

The significance of the innate immune system in early OA becomes evident, as it leads to direct chondrocyte and cartilage destruction as well as NF*κ*B activation with pronounced redundancy and perpetuation. As stated previously, NF*κ*B is the master regulator of the immune response. It is involved in the activation of complement, defensins, adhesions, and caspase-1, as well as the production of cytokines, reactive oxygen species (ROS), and NO. Despite the attractiveness of targeting NF*κ*B in disease-modifying therapy, it is an unreliable target in large part due to its universal role in normal cellular signaling. Its modulation has a significant side effect profile; however natural health products such as those found in grapes and green tea have shown promise but need further study [67].

**Figure 4:**
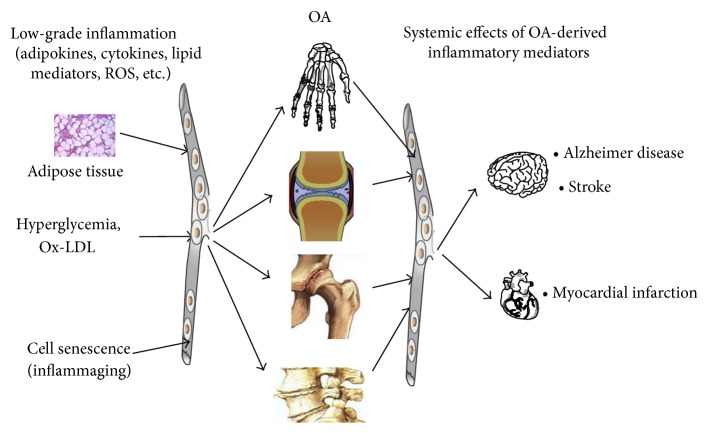
Model for role of systemic proinflammatory state and OA. Inflammatory mediators released into blood enter the joint exacerbating OA, which releases its own mediators of inflammation leading to increased systemic inflammation (reproduced with permission from Berenbaum [6]).

The difficulty in treating OA is that once local and systemic inflammation is established, debilitative changes in affected joints are difficult to control. OA is both affected by and contributing to a baseline proinflammatory state, such as that seen in senescence, metabolic syndrome, and Alzheimer's disease amongst others (Figure 4) [4]. For example, Berenbaum et al. found that a high fat diet increased inflammation in the acute phase of OA [7]. In another study, Kyrkanides et al. found that inducing OA in mice genetically susceptible to Alzheimer's disease exacerbated and accelerated neuroinflammation, increasing the number and size of amyloid plaques [68]. Many therapies, including anti-TNF*α* and anti-IL-1*β* therapy, have been shown to decrease inflammation but fail to significantly improve function or prognosis in established OA patients [3, 4]. Pain levels have been shown to have a statistically significant correlation with level of change in synovitis (*r* = 0.21, *P* = 0.0003), but not cartilage destruction or baseline level of synovitis [69]. This correlation is only modest and does nothing but supporting the notion that a relative increase in inflammation will increase perception of pain. There is a disconnection between biomarkers of disease, radiographic change, and symptomology, complicating treatment. Degenerative change in OA can occur under two months following trauma [70], and epigenetics has been shown to play a role in mediating the acute inflammatory changes driven by the altered joint microenvironment [71]. It is for these reasons that we hypothesize that addressing early inflammatory change in the synovium consistent with OA is crucial in modulating disease progression and therefore patient disability. Therapies that have failed to show benefit to date may be effective when implemented at an appropriate stage of disease. Future research should be targeted toward identifying at-risk patients and early intervention.

## 4. Perspectives

Pharmacological treatment to date has had varying effects on symptomology, but disease modulation has yet to be attained. Common modalities include NSAIDS, corticosteroids, chondroitin sulfate, and glucosamine [72]. These treatments are variably effective on an individual basis and often only provide temporary relief and are needed to be repeated chronically. Trials of anti-TNF*α* and anti-IL-1*β* therapy for disease modulation have been unsuccessful despite the dominance of TNF*α* and IL-1*β* in pathogenesis [4]. Chevalier et al. concluded that IL-1*β* antagonism may benefit patients with baseline high levels of pain if administered in low, frequent intra-articular (IA) injections to avoid neutropenia and serious infection [73]. One* in vivo* study revealed that IA injection of lubricin up to two weeks after injury reduced severity of OA in mice, while local antioxidants such as N-acetylcysteine after injury showed promise* in vitro* [74, 75]. The proposed benefit of these treatments administered soon after injury in injury-induced OA underlines the significance of early intervention in OA pathogenesis. Treatment with fibroblast growth factor 18, which is specific for the anabolic FGFR-3 versus the catabolic FGFR-1, is currently on trial [71].

We believe that regular screening is needed and is justified as OA is ubiquitous in seniors aged over 65, is clinically present in 13.9% of US adults aged 25 or above, and is a leading cause of disability and hospitalization in the USA [1, 2, 72]. However, further studies are needed in order to establish guidelines for screening. We recommend that regular screening for OA be implemented on an outpatient basis. Special attention should be given to patients of 65 years or above and patients with metabolic syndrome, Alzheimer's disease, or other systemic proinflammatory states. Candidate markers for screening should continue to be researched with particular attention paid to local articular levels. IL-6, complement, and ratio of FGFR-3/FGFR-1 should be considered.

Additionally, the role of physical, imaging, or combination diagnostic paradigms must be considered. Contrast-enhanced MRI and power Doppler ultrasound are the leading imaging modalities for synovitis [46]. Identifying early and specific changes in OA may best be visualized using PET, NIRF, or SPECT imaging. Many probes for cells and proteins involved in OA pathogenesis are listed in Table 3 and Figure 3. We are currently developing a Tc99m-cFLFLF/SPECT technique to visualize early leukocyte recruitment in OA joints based on a preliminary* in vivo* study (Figure 2). While our probe is not 100% specific for leukocytes, we are currently in the process of identifying more specific receptors.

As changes in the joint consistent with OA can occur rapidly following injury and are associated with inflammation, intervention should be aimed at the early reactive phase of OA pathogenesis [70]. Importantly, past therapeutic trials may have failed due to attempted intervention at irreversible stages of disease. Wang et al. showed that knocking out components of the complement cascade greatly reduced incidence of OA in mice [17] and this strategy for treatment management should be further researched. A study assessing whether there is an increased relative risk of OA diagnosis and severity in patients with paroxysmal nocturnal hemoglobinuria could be beneficial in this regard. Anticomplement therapy should initially be attempted locally to narrow the focus of treatment and lower the incidence of potential severe infection.

While disease modification in OA still eludes the medical community, recent advances in pathogenesis and understanding of the disease process beseech hope to solving the riddle of a ubiquitous, costly disease that significantly diminishes quality of life in millions of patients. With guided further research and international collaboration, we believe that early detection and intervention in OA are possible. Due to the lack of success and discrepancy of disease modulation between* in vivo* and* in vitro* studies, the significance of identifying patients in the early phase of disease becomes paramount in experimenting with detection and treatment of the disease process. Screening must be implemented in high-risk patients, and early, aggressive treatment is necessary and mandated to avoid substantial morbidity.

## Figures and Tables

**Table 1:** Role of the essential cells implicated in OA pathogenesis.

Cell type	Role	Comments
Macrophages	(i) Line intimal layer [5, 14] (ii) Required for production of MMPs and cartilage damage [5, 14] (iii) TNF*α*, IL-1, MMP, TGF*β*, IL-10, IL-12, and chemokine production [29] (iv) Mediators of TGF*β* induced osteophyte formation [76] (v) Possess TLRs [14]	

T cell (TCR = T-cell receptor)	(i) Line subintimal layer [5] (ii) Present in different stages of activation: early (CD69^+^), intermediate (CD25,38^+^), and late (CD45RO^+^) [34]^ ^ (iii) Increased CD4^+^/CD8^+^ ratio in OA knees versus control [35] (iv) Th1 > Th2 subset, as well as increased Th1 cytokine product IFN*γ* [34, 36] (v) Increased CD3*ε* ^+^ T cells and CD3*ε* ^+^/CD3*ζ* ^+^ T cell ratio in OA synovium and decreased ratio of CD3*ζ* ^+^ T cells [65] (vi) Produce chemokines attractant to macrophages [65] (vii) CDR3 similarity in TCR [77] (viii) Several autoantigens including those on chondrocyte membrane have been identified [31, 78, 79] (ix) Cartilage linking protein and G1 domain of aggrecan have shown promise as potential autoantigens to follow [31, 78, 79]	(i) Suggestive of chronic inflammation (ii) Suggestive of oligoclonal expansion and antigen-driven response

Mast cell (MC)	(i) Numbers are at least as high as those in RA synovium [5] (ii) Mostly in subintimal layer and around blood vessels [22] (iii) Levels positively correlated with total cellular infiltrate, however no correlation with ESR [22] (iv) Degranulated MCs found most commonly in intimal layer [23] (v) Higher ratio of tryptase to tryptase/chymase phenotype in OA than controls [24] (vi) Selective expansion of tryptase MC phenotype [24]	(i) MCs lie around blood vessels and mediate vascular permeability hinting at crucial role of MCs, however not related to ESR and degranulated phenotype seen in intimal layer (ii) Tryptase phenotype suggestive of degranulation

B cells	(i) Not always present or may be present in small numbers [24] (ii) Mostly in subintimal layer [5] (iii) Undergo oligoclonal expansion with similar CDR3 regions [80, 81] (iv) Evidence of somatic hypermutation [80, 81] (v) In patients with moderate to strong infiltration, there were presence of germinal centers and increased T-cell populations [80, 81] (vi) Potential role as antigen presenting cell [80, 81] (vii) Several autoantigens have been identified including those on the chondrocyte membrane but multiple antigens have been discovered and patterns have yet to be characterized [31] (viii) Elevated antibody titer to cartilage membrane in OA patients versus control [82] (ix) Secrete IL-6 [83]	(i) Suggestive of antigen-driven response

Fibroblast	(i) Activated by both IL-1*β* and TNF*α* and must neutralize both to decrease activation [29] (ii) Produces MMPs, IL-6, IL-8, ADAMTS-4,5, and MCP-1 [29]	

NK cell	(i) May have role in early pathogenesis of OA: found to have CD16^+^CD56^+^ phenotype [19] positive for granzymes A and B [19, 21] and CD16^−^CD56^+^ phenotype negative for granzymes A and B [20] (ii) Poor *in vitro* IFN*γ* production upon stimulation in late OA patients [20] (iii) Stimulated by IL-15 [66]	(i) Suggestive of activation/exhaustion phenotypes

Neutrophil (PMN)	(i) Generally not found in OA synovial tissue, but sometimes present [5] (ii) HNP1-3 (mainly produced by PMNs) found in synovia of OA patients, with levels inhibited by TNF*α* stimulation [27] (iii) NGAL (mainly produced by PMNs) found in OA synovia complexed with MMP-9, decreasing its degradation and increasing glycosaminoglycan levels released from cartilage explants [27]	(i) PMNs may play a role in the earliest stages of OA and therefore might not be expected to be identified in most studies of established OA samples

**Table 2:** Role of dominant effectors involved in OA pathogenesis.

Protein	Role	Comments

IL-1*β*	(i) Produced by macrophages [29] (ii) Receptors upregulated in OA chondrocytes and fibroblasts [84] (iii) Stimulates production of MMPs [85], ADAMTS-4 [86], and chemokines [87] (iv) Inhibits proteoglycan and type II collagen via repressing GlcAT-1 [88] (v) Induces apoptosis in chondrocytes via upregulation of Bcl-2 family of proteins, mitochondrial depolarization [89], and perhaps ROS [90] and NO production [91]	(i) GlcAT-1 is an important enzyme for production of glycosaminoglycan

TNF*α*	(i) Produced by macrophages [29] (ii) Promotes resorption and inhibits production of proteoglycan in cartilage [30] (iii) Stimulates MMP and chemokine production [32] (iv) Decreases collagen production [32] (v) May form a negative feedback loop with HNP1-3 [25]	

IL-6	(i) Produced by fibroblasts [14], chondrocytes [92, 93], and B cells [83] (ii) Production by chondrocytes induced by PGE_2_ [92], TNF*α*, and IL-1*β* [93] (iii) Found to be present in intimal layer and produced mostly by plasma cells when detected in high levels (>600 pg/mL) in synovial fluid [83] (iv) Activates JAK/STAT to inhibit aggrecan core and link protein and type II collagen gene expression; blocking STAT phosphorylation inhibits this downregulation [94] (v) After binding to its receptor, it binds and inactivates transcription factor for COL2A1 gene, which encodes procollagen chain of triple helix of type II collagen [95] (vi) Upregulates expression of MMPs in conjunction with IL-1 [96]	

Complement	(i) Expression and activation abnormally high in OA synovium, significantly in early OA [17] (ii) MAC present around chondrocytes and in synovium in late OA [17] (iii) MAC stimulates MMP, ADAMTS, and chemokine production in chondrocytes [17] (iv) Cartilage ECM, fibromodulin, and aggrecan induced formation of C5b-9 [17] (v) C5^−^ knockout mice showed no significant synovitis or cartilage loss versus control C5^+^ mice 8–12 weeks s/p medial meniscectomy [17] (vi) C6^−^ mice developed roughly half the degeneration from synovitis as C6^+^ mice s/p medial meniscectomy [17] (vii) CD59^−^ mice developed more severe OA [17] (viii) C1s cleaves IGFBP-5 which is chondroprotective [18] (ix) C1s inhibition shown to promote better joint architecture in dogs [18]	

TLR	(i) Activated by DAMPs released from ECM in joint damage [14] (ii) Induce proinflammatory cytokine production (IL-1*β*, TNF*α*, MMP, etc.) by macrophages [14] (iii) Induce catabolic pathways in chondrocytes [15] (iv) Upregulated on chondrocytes in advanced OA [15] (v) TLR4 on OA chondrocytes more sensitive to S100 than control [16]	(i) S100 is a DAMP

PGE_2_	(i) Upregulated in OA joints [92] (ii) Inhibits proteoglycan synthesis by suppressing aggrecan gene transcription [92] (iii) EP2,4 receptors upregulated in joint cartilage as OA progresses [92] (iv) Decreases collagen type II/type I ratio [92] (v) When coupled with IL-1 stimulation, it greatly increases expression of IL-6 and iNOs [92]	

ADAMTS	(i) ADAMTS-4 can be downregulated by inhibiting TNF*α* and/or IL-1*β* while ADAMTS-5 is constitutive in human [29]	(i) Uncertainty over which of the two is more significant in OA pathogenesis

TGF*β*	(i) Osteophyte formation [76]	

VEGF	(i) Promotes angiogenesis and MMP production [14]	

IL-4,7,8,10,13,15,17,18, adipokines, and leukemia inhibitory factor	(i) Detected in synovium [5] (ii) IL-17 works synergistically with TNF*α* and IL-1 and is released by mast cells [97] (iii) Increased levels of IL-15 in early versus late OA [66]	

**Table 3:** Probes for mediators of inflammation in modern imaging techniques.

Cell type or protein	Probe
Macrophage	(i) ^18^F-FDG (PET) [98, 99]

CD4^+^ T cell	(i) ^64^Cu-PTSM (PET) [100] (ii) ^18^F-FB-IL-2 (PET) [99]

B cell	(i) ^124^I-rituximab (PET) [101]

Neutrophil	(i) cFLFLF-PEG-Cy 7 (NIRF) (ii) cFLFLF-PEG-DOTA-^64^Cu (PET)

Mast cell	(i) Ligand 1 (*in vitro*) [102]

TNF*α*	(i) ^64^Cu-DOTA-etanercept (PET) [103]

Complement	(i) USPIO-conjugated anti-C3mab (T2-MRI) [104]

MMP	(i) ^124^I-HO-MPI (CGS 27023A) (PET) [105]
